# Gaze-Probe Joint Guidance with Multi-task Learning in Obstetric Ultrasound Scanning

**DOI:** 10.1016/j.media.2023.102981

**Published:** 2023-12

**Authors:** Qianhui Men, Clare Teng, Lior Drukker, Aris T. Papageorghiou, J. Alison Noble

**Affiliations:** a Institute of Biomedical Engineering, Department of Engineering Science, University of Oxford, Oxford, OX3 7DQ, United Kingdom; b Nuffield Department of Women’s & Reproductive Health, University of Oxford, Oxford, OX3 9DU, United Kingdom; c Department of Obstetrics and Gynecology, Tel-Aviv University, Tel Aviv, Ramat Aviv, 69978, Israel

**Keywords:** Fetal ultrasound, Probe guidance, Multimodal representation learning, Multi-task learningo

## Abstract

In this work, we exploit multi-task learning to jointly predict the two decision-making processes of gaze movement and probe manipulation that an experienced sonographer would perform in routine obstetric scanning. A multimodal guidance framework, *Multimodal-GuideNet*, is proposed to detect the causal relationship between a real-world ultrasound video signal, synchronized gaze, and probe motion. The association between the multi-modality inputs is learned and shared through a modality-aware spatial graph that leverages useful cross-modal dependencies. By estimating the probability distribution of probe and gaze movements in real scans, the predicted guidance signals also allow inter- and intra-sonographer variations and avoid a fixed scanning path. We validate the new multi-modality approach on three types of obstetric scanning examinations, and the result consistently outperforms single-task learning under various guidance policies. To simulate sonographer’s attention on multi-structure images, we also explore multi-step estimation in gaze guidance, and its visual results show that the prediction allows multiple gaze centers that are substantially aligned with underlying anatomical structures.

## 1 Introduction

Ultrasound (US) scanning is a real-time, radiation-free inner-body monitoring procedure that has become the primary modality in obstetrics for pregnancy screening and diagnosis. However, operating the US machine requires refined hand-eye co-ordination of a scanner to read an image and manipulate a handheld probe simultaneously, which makes it a highly-skilled medical examination. Computer-assisted scanning with probe motion guidance has been increasingly investigated among researchers and clinicians (Housden et al., 2008; Prevost et al., 2018; Grimwood et al., 2020; Guo et al., 2020; Zhao et al., 2022) to improve the training process for non-specialists. One of the main goals in this field is to estimate probe positional parameters for freehand 3D US reconstruction or plane-based localization. Prevost et al. (2018) reconstructed US volumes from convolutional neural network (CNN) integrated with inertial measurement unit (IMU) motion signals. Recently, Zhao et al. (2022) considered a hybrid architecture to improve probe motion estimation by detecting local key points in US biometric planes. Within the robotics field, work has focused on guiding operators to scan simple structures such as the liver (Mustafa et al., 2013), lumbar and vertebrae (Li et al., 2021). Such solutions targeting fixed structures or tissues are not feasible for obstetric scans due to the variety of fetal anatomy to be measured and uncontrollable fetal movement in the womb.

In obstetric scanning guidance, a common practice is to treat probe guidance as an image-guided navigation problem. For example, Di Vece et al. (2022) regressed six-dimensional pose of the US head plane relative to the center of fetal brain using phantom data. Zhao et al. (2021) proposed to position the probe based on landmark-based image retrieval. Other work in this field (Toporek et al., 2018; Wang et al., 2019) deployed probe guidance signals to a robotic arm that is not practically applicable in a clinical environment. Regarding the multi-modality guidance, Droste et al. (2020b) simulated the next probe movements based on the motion guidance in previous steps using a behavioral cloning system. In Droste et al. (2020b), different policies are also modeled for operators to either follow the next-action instruction or directly approach the anatomical Standard Plane (Baumgartner et al., 2017). As observed in our previous work (Men et al., 2022), the visual focus on the US image will also influence the sonographer of how to move the probe in the following steps. Hence, the eye gaze can provide instructive localization about consequent probe movements.

Other than probe navigation, gaze information is also used as a guidance signal, usually in the form of gaze-point or saliency map (eye-tracking heat map) prediction from US image or video. Cai et al. (2018a,b) leveraged visual saliency as auxiliary information to aid abdominal circumference plane (ACP) detection, and Droste et al. (2019) extended it to various anatomical structures which is more applicable in real-time scanning guidance. Teng et al. (2021) characterized the visual scanning patterns from normalized time series scanpaths. Since a sonographer will act accordingly to the next image resulting from their hand movement on probe, the main objective of the current paper is to learn the correspondence between visual attention and probe motion, and to explore how they are guiding each other during an US scan.

In our earlier work (Men et al., 2022), we proposed a first model to provide step-by-step guidance in both synchronized probe and gaze signals to the desired anatomical plane. In the data acquisition procedure, we recorded scanning patterns of experienced sonographers by collecting a large number of real-world probe motion, gaze trajectory, and US videos from routine obstetric scanning. The proposed multi-modality network (Multimodal-GuideNet) employs multi-task learning (MTL) for the two highly-related US guidance tasks of probe motion prediction and gaze trajectory prediction, and identifies commonalities and differences across these tasks. The performance boost over single-task learning models suggests that jointly learning gaze and probe motion leads to more objective guidance during US scanning. Moreover, the model generates real-time probabilistic predictions (Graves, 2013) that provide unbiased next-step guidance of the two signals to aid operators.

As an extension of Men et al. (2022), in the current paper we present a more detailed elaboration of the motivation and the methodology of how to provide real-time scanning guidance by modeling the inter-dependency between the synchronized multimodal signals. Other than the stepwise guidance presented in Men et al. (2022), we consider an additional application of global probe guidance directly toward the target biometric view, and additional experiments are conducted to show the generalizability of adopting gaze information to help probe guidance under different guidance policies. In terms of gaze, we extend the previous policy of next-step gaze shift prediction to the multi-step gaze center prediction with a Gaussian Mixture Model (GMM) as the probabilistic distribution (Zhao and Wildes, 2021), which reveals multiple key areas in the ultrasound image that the sonographer may be interested in the following scanning steps. Saliency map-based evaluations are also performed to show the adherence between the statistics of predicted areas and the anatomical structures as clinical explainability to support the approach.

In summary, we have significantly expanded the preliminary work (Men et al., 2022) with the following contributions: 1) In probe guidance, we extend the human-like stepwise guidance to the task of global guidance toward the target in a single movement. 2) In gaze guidance, a new application of predicting multiple gaze focuses of sonographers in the next few steps is proposed with anatomical relevance. 3) Extensive experiments based on saliency metrics are conducted for gaze prediction and its statistical analysis with clinical explanations, and an additional sensitivity test is performed to assess the robustness of the proposed multimodal system. 4) We include a comprehensive discussion of the clinical significance in predicting the causal relationship between the ultrasound multi-modality signals.

## 2 Method

**Fig. 1:**
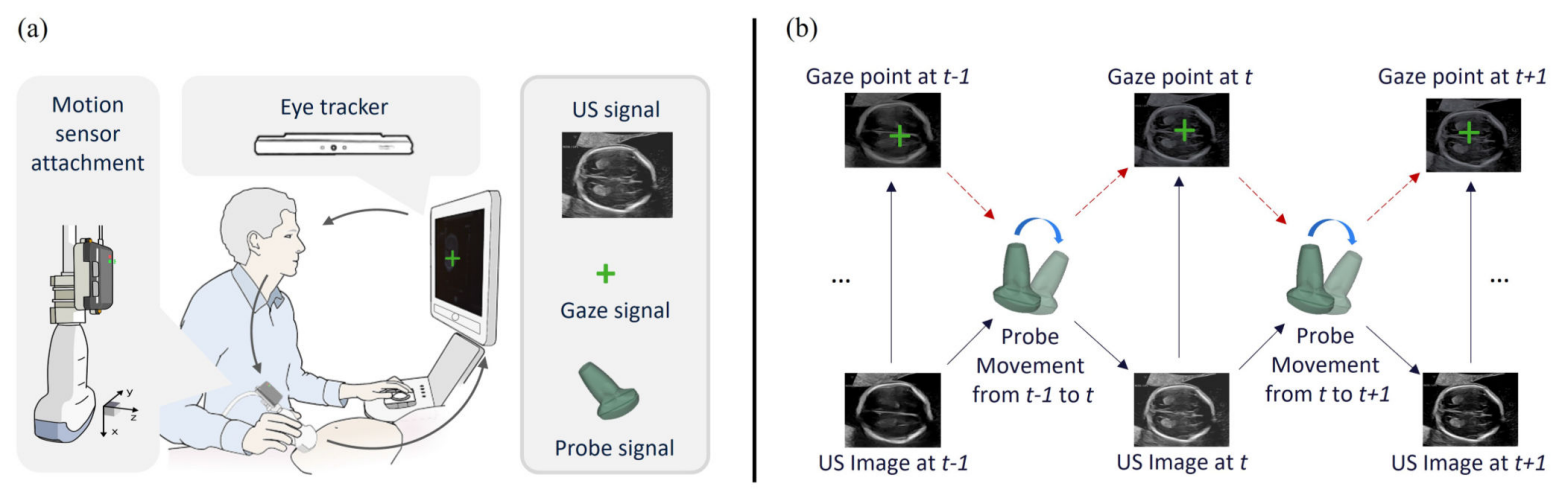
Data acquisition and the causality between captured signals. (a) Overview of the multi-modality data acquisition in the clinical obstetric ultrasound scanning. (b) The stepwise cause and effect between the acquired probe motion signal, the gaze signal, and the US image.

Figure 1 outlines the principles of the approach. The probe orientation is recorded in 4D quaternions by an IMU motion sensor attached to the US probe, and the 2D gaze-point signal is captured by an eye-tracking sensor mounted on the bottom of the US screen. Given an US image ***I**_t_* starting at a random plane, its change in gaze ***s**_t_* between neighbour time steps, and its corresponding probe rotation *r_t_*, the proposed multi-task model Multimodal-GuideNet estimates the instructive movements of both the gaze and probe for the standard plane acquisition. The two main tasks: probe motion prediction and gaze shift prediction complement each other in US scanning guidance. The task policies, problem definition, and network architecture are presented next.

### 2.1 Policies and problem formulation

Unlike previous US guidance models that only predict a fixed action, we regard the gaze and probe movements as random variables to account for inter- and intra-sonographer variations. This enables an extendable visual region for gaze focus and a flexible physical area for probe placement that simulates real-world US scanning.

We explore two separate policies for gaze estimation: First, we predict the next-step gaze movement under the observation of the current US image and probe signal. The policy is denoted as one-step gaze prediction (OG) which retrieves the most plausible area that the sonographer is likely to focus on. However, since there will normally be more than one anatomical structure in an US plane (Sharma et al., 2021; Droste et al., 2020a), we consider a second policy of multi-step gaze prediction (MG), which aims to predict multiple potential areas that an expert sonographer may be interested in by estimating the gaze centers in the next several steps.

For probe movement prediction, we also provide guidance signals towards two different navigation targets, i.e., the next movement of the probe for one-step rotation prediction (OP) and the movement for standard plane orientation prediction (SP). The OP policy aims to closely imitate the action of a human sonographer when performing routine obstetric scanning, and the SP policy is more suitable to be deployed for a direct standard plane acquisition.

#### 2.1.1 Gaze

##### One-step gaze shift prediction (OG)

Let *s_t_* = *g_t_* - *g_t-1_* be the shift of a gaze point *g* = (*x, y*) at the single timestamp *t*. We assume that the gaze shift *s_t_* at time *t* follows a bi-variate Gaussian distribution, i.e., $s_{t}∼\;N(\mu_{t}^{OG},\sigma_{t}^{OG},\rho_{t}^{OG}),$ where $\mu_{t}^{OG}$ and $\sigma_{t}^{OG}$ denote the mean and standard deviation respectively in 2D, and $\rho_{t}^{OG}$ is the correlation coefficient between *x* and *y*. Therefore, at every step, the model outputs a 5-dimensional vector for gaze estimation. The probability function for one-step gaze shift is defined as ${\mathbb{P}}_{s}^{OG}=\mathbb{P}(s_{t}|\mu_{t}^{OG},\sigma_{t}^{OG},\rho_{t}^{OG}).$

##### Multi-step gaze center prediction (MG)

Since single-step gaze prediction is stochastic, we also explore a second application to predict gaze centers in the next few steps. This is done by clustering the gaze points in a sliding time window of *F* steps into *L* clusters, of which the cluster centers reflect the main gaze fixations of the sonographer on each ultrasound image. Let $s_{t}^{l}=c_{t}^{l}-c_{t-F}^{l}$ be the displacement of the l-th gaze center $c^{l}=(x_{c}^{l},y_{c}^{l})$ within *F* timestamps. Here, we make the assumption that the multi-step gaze displacement follows a bivariate Gaussian Mixture distribution with *L* components, i.e., $s_{t}∼\sum_{l=1}^{L}\pi_{l}N(\mu_{t}^{MG}(l),\sigma_{t}^{MG}(l),\rho_{t}^{MG}(l)),$ where $\mu_{t}^{MG}(l),\sigma_{t}^{MG}(l),$ and $\rho_{t}^{MG}(l)$ are Gaussian parameters for the *l*-th center, and the weight *π_l_* is its probability with $\sum_{l=1}^{L}\pi_{l}=1.$ With the component weight *π_l_*, each predicted center will show different importance learned from the real gaze distribution. Similar to the single-step setting, the multi-step gaze estimation outputs a 6 × *L* dimensional vector including the component weights. The probability function for multi-step gaze distribution is thus given as ${\mathbb{P}}_{s}^{MG}=\sum_{l=1}^{L}\pi_{l}\mathbb{P}(s_{t}|\mu_{t}^{MG}(l),\sigma_{t}^{MG}(l),\rho_{t}^{MG}(l)).$

#### 2.1.2 Probe

##### One-step probe prediction (OP)

We first estimate the next probe rotation that the sonographer would perform. The purpose of this policy is to imitate the step-by-step scanning process of an expert sonographer. Let $r_{t}=q_{t-1}^{*}q_{t}=q_{t-1}^{t}$ be the rotation from the probe orientation q = *(q_w_, q_x_, q_y_, q_z_)*, where q^*^ is the conjugate. We thus achieve a 14-dimensional vector for probe rotation *r_t_* including the parameters of mean $\mu_{t}^{OP}$ and a 4 × 4 covariance matrix $\sum_{t}^{OP}$ across the four quaternion parameters. Similar to the one-step gaze dynamics, we assume that the probe rotation *r_t_* at time *t* follows a multi-variate Gaussian distribution $r_{t}∼\;N(\mu_{t}^{OP},\sum_{t}^{OP}),$ and the corresponding probability function is defined as ${\mathbb{P}}_{r}^{OP}=\mathbb{P}(r_{t}|\mu_{t}^{OP},\sum_{t}^{OP}).$

##### Standard plane orientation prediction (SP)

At every step, we also consider estimating the rotation towards the standard plane. This policy is optimal if the final standard plane is straightforward to reach with an explicit orientation. Here, the probe rotation *r_t_* becomes $q_{t-1}^{*}q_{T}$ that is the rotation from probe orientation at time *t-*1 to the standard plane at time *T*. Accounting for different sonographer’s action, the goal rotation *r_t_* under the new policy is also assumed to be multi-variate Gaussian distributed with the probability denoted as ${\mathbb{P}}_{r}^{SP}=\mathbb{P}(r_{t}|\mu_{t}^{SP},\sum_{t}^{SP}).$

#### 2.1.3 Training loss

We jointly minimize the negative log-likelihoods of the two learning tasks as the multi-task objective function

(1) $$ℒ=\sum_{t=t_{0}}^{T}(-\lambda_{s}\log{\mathbb{P}}_{s}-\lambda_{r}\log{\mathbb{P}}_{r}+\;\eta {\left(1-∥\mu_{t}^{r}{∥}^{2}\right)}^{2}),{\mathbb{P}}_{s}\in \{{\mathbb{P}}_{s}^{OG},{\mathbb{P}}_{s}^{MG}\},{\mathbb{P}}_{r}\in \{{\mathbb{P}}_{r}^{OP},\;{\mathbb{P}}_{r}^{SP}\}$$

where *t_0_* and *T* are the start and end indices for prediction, respectively. *λ_s_* and *λ_r_* control the training ratio of the two tasks. From the same range of log-likelihood, the two weights are both set to 1. η is the weighting parameter for the quaternion prior to normalize $\mu^{r}\in \{\mu^{OP},\mu^{SP}\}$ with *η* = 50.

### 2.2 Multimodal-GuideNet

**Fig. 2:**
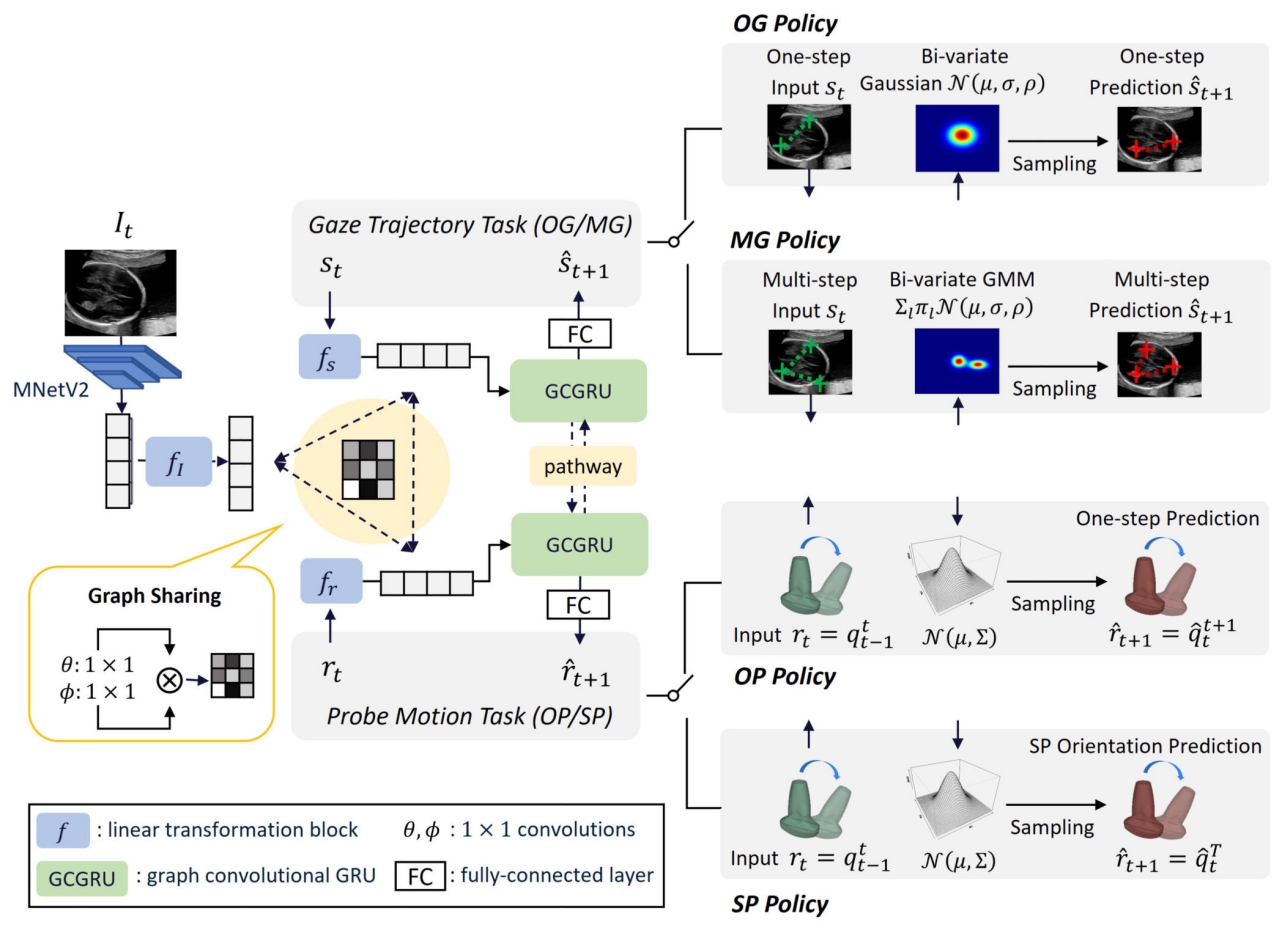
Flowchart of Multimodal-GuideNet. The tasks of gaze trajectory prediction and probe motion prediction share a modality-aware spatial graph from the three modalities. The predictions in OP (one-step probe rotation prediction) and SP (standard plane orientation prediction) policies follow multivariate Gaussian distributions. The predictions in OG (one-step gaze shift prediction) policy follows bi-variate Gaussian distribution, and in MG (multi-step gaze center prediction) policy follows Gaussian Mixture distribution.

The multimodal guidance framework is presented in Fig. 2. Starting from the left of the figure, Multimodal-GuideNet constructs a lightweight graph shared among the three modalities to facilitate multi-task learning. The network backbone is formed by a graph convolutional Gated Recurrent Unit (GCGRU) (Li et al., 2016) that automatically allocates useful dependencies between the three modalities at each guidance step for structured sequence modeling. Spatially, the designed lightweight graph consists of one-layer graph convolution that is computationally efficient for online inference. Temporally, the gaze and probe signal each pass through a GRU (Cho et al., 2014) to process the individual dynamics, which allows real-time guidance with arbitrary length of input. Inside the GRU, the two dynamics exchange hidden states to complement each other through a bidirectional pathway. Finally, GRU outputs the predicted distributions of the two-modality signals based on the predefined policies shown on the right of Fig. 2.

To learn the feature representation of the three signals, the grayscale US image is initially encoded into a 640-channel 7 × 9 feature map with MobileNetV2 (MNetV2) (Sandler et al., 2018), and then pooling and flattening into a 1,920-channel vector as the image representation input as in Droste et al. (2020b).

To facilitate interactive learning within the graph structure, the input of image feature, probe, and gaze movement is embedded into an equal-sized 128-channel vector separately through a linear transformation block *f_I_*, *f_s_*, and *f_r_*, each of which contains a fully-connected (FC) layer, a batch normalization (BN) layer, and a ReLU activation function.

#### 2.2.1 Modality-aware graph representation sharing

To learn the inter-modal interactions, we propose a graph structure *G_t_* = (*𝒱_t_*, *S_t_*) that is shared among the three modalities at each time t, where *𝒱_t_* = {*f_I_*(*I_t_*), *f_s_*(*s_t_*), *f_r_*(*r_t_*)} is the vertex set with 3 nodes. *ε_t_* is the edge set connecting any two nodes in *𝒱_t_*. The connection strength within *ε_t_* is specified by a 3 × 3 adaptive adjacency matrix $A_{t}=\;A^{\omega }{}_{t}+M_{t}.$ The first term $A_{t}^{\omega }$ indicates the intrinsic spatial proximity of the multi-modality graph *G_t_*, with each entity $A_{t}^{\omega }(j,k)$ supervised by the corresponding two modality features *f_j_*(*j_t_*) and *f_k_*(*k_t_*) in the embedded space:

(2) $$A_{t}^{\omega }(j,k)=softmax(\theta {\left(f_{j}(j_{t})\right)}^{T}\phi (f_{k}(k_{t}))),\;j,k\;\in \;\{I\;,\;s,\;r\}$$

where *θ* and *φ* are 1 × 1 convolutions (Zhang et al., 2020) with *θ(x)*, *φ(x)* ∈ ℝ^256^, and the *softmax* operation is to normalize the row summation of $A_{t}^{\omega }.$ As the second term in *A_t_*, *M_t_* is a learnable adjacency mask (Yan et al., 2018) to increase graph generalization. The parameters in *M_t_* are not constrained but trained together with the other parameters in the model, which provides flexibility to the multi-modality graph. In general, at a time step *t*, $A_{t}^{\omega }$ reflects the explicit connectivity between modalities, while *M_t_* is target-oriented with the implicit connection driven by the signal guidance objective.

The multi-modality features are then integrated by the graph representation defined in *A_t_* and transferred to the probe and gaze signals. Specifically, the message passed for the gaze movement *s* and probe movement ***r*** is aggregated by a spatial graph convolutional layer with *sigmoid* activation among the three modalities

(3) $$\sum_{k\in \{I,s,r\}}\frac{1}{1+\exp(-A_{t}(j,k)f_{k}(k_{t})W_{j})},\;j\in \{s,\;r\}$$

here *W_j_* is the input feature kernel specified for each gate in the GRU cell, which will reconstruct the two-modality signals for the upcoming time step(s).

#### 2.2.2 Gaze-probe bidirectional pathway

During US scanning, the gaze and probe movements are generally heterogeneous, i.e., they do not move at the same pace. The gaze contains rapid eye movements between anatomical structures upon approaching the standard plane, while the probe remains steady. We account for this effect by enclosing a bidirectional inverse adaptive *pathway* between the hidden states of *s_t_* and *r_t_*.

We denote $h_{t}^{s},$, $h_{t}^{r}$ and ${\tilde{h}}_{t}^{s},{\tilde{h}}_{t}^{r}$ as the hidden state and candidate activation, and $z_{t}^{s},$ $z_{t}^{r}$ as the update gate of GRU for gaze s and probe ***r***, respectively. Under a single signal source, the hidden state *h_t_* is updated by $(1-z)⊙h_{t-1}+z_{t}⊙\tilde{h_{t}},$ and the candidate activation $\tilde{h_{t}}$ is updated by *tanh(W_h_x_t_* + *U_h_(γ_t_Θ**h**_t-1_)+b_h_)*, where *γ_t_* is the reset gate, and *W_h_*, *U_h_*, and *b_h_* are the parameters for hidden state h as defined in a standard GRU (Cho et al., 2014). Here, with the bidirectional adaptive pathway, each of the two highly-related signals s and r will be adjusted by not only its own state, but also the state of the other signal. The hidden states of these two guidance signals are defined by:

(4) $$h_{t}^{s}=\underset{\;\text{update}\;\text{from}\;\text{gaze}\;}{\underset{︸}{\alpha (1-z_{t}^{s})⊙h_{t-1}^{s}+\alpha z_{t}^{s}⊙\tilde{h_{t}^{s}}}}+\underset{\;\text{inverse}\;\text{update}\;\text{from}\;\text{probe}\;}{\underset{︸}{(1-\alpha )z_{t}^{r}⊙h_{t-1}^{s}+(1-\alpha )(1-z_{t}^{r})⊙\tilde{h_{t}^{s}}}}$$

(5) $$h_{t}^{r}=\underset{\;\text{update}\;\text{from}\;\text{gaze}\;\text{probe}\;}{\underset{︸}{\beta (1-z_{t}^{r})⊙h_{t-1}^{r}+\beta z_{t}^{s}⊙\tilde{h_{t}^{r}}}}+\underset{\;\text{inverse}\;\text{update}\;\text{from}\;\text{gaze}\;}{\underset{︸}{(1-\beta )z_{t}^{s}⊙h_{t-1}^{r}+(1-\alpha )(1-z_{t}^{s})⊙\tilde{h_{t}^{r}}}}$$

where *α*, *β* are the adaptive channel-wise weights for $z_{t}^{r},$ respectively, and ⊙ is element-wise product. The number of hidden channels is set to 128 which is the same as *α* and *β*. With the proposed bidirectional pathway, the gaze and probe signals will adapt the domain-specific representation from each other to generate a more accurate scanning path. Other than the input operation for all gates (Eq. 3) and an adaptive hidden state (Eq. 4, 5) for the output, we follow the operations in the standard GRU (Cho et al., 2014) to transfer temporal information. For each of the two signals, the updated hidden state is eventually fed to a fully-connected layer that outputs the probabilistic parameters based on the given policy.

## 3 Experiment

### 3.1 Multi-modality obstetric scan

The ultrasound video, eye gaze, and probe motion data used in this study were acquired from the PULSE (Perception Ultrasound by Learning Sonographic Experience) project (Drukker et al., 2021). The clinical fetal ultrasound scans were conducted on a GE Voluson E8 scanner (General Electric, USA) and the video signal was collected lossless at 30 Hz. The synchronized gaze tracking data was recorded with a Tobii Eye Tracker (To-bii, Sweden) as 2D coordinates (*x, y*) at 90 Hz. The probe motion was simultaneously recorded with an IMU (x-io Technologies Ltd., UK) attached to the probe cable outlet as shown in Fig. 1(a). For this study, approval from the UK Research Ethics Committee was obtained and written informed consent was given by all participating pregnant women and sonographers. In total, there are 551 *Anomaly* (2^nd^ trimester) and *Growth* (3^rd^ trimester) scans carried out by 17 qualified sonographers. All three-modality data were downsampled to 6 Hz to reduce the time complexity while preserving the temporal properties of the scans.

### 3.2 Implementation details

The standard plane acquisition is to choose a clinical standard view which is usually one step before freezing the US video for annotation (Sharma et al., 2021), and the freeze state is recognised automatically by Optical Character Recognition (OCR) (Kay, 2007). For each acquisition, a multimodal data sample is selected 10 seconds (10s) before the standard plane, which is the time for probe refinement (Droste et al., 2020b). In total, there are 2,121 eligible video clips extracted from all acquisitions, among which 1,681 clips are used for training and the rest for testing. We also discard irrelevant graphical user interface information from the video clip, and perform data augmentation by randomly adjusting the brightness and contrast of the video frames within 10% range, and randomly cropping within 20% of the image size. The augmented images were then resized to 224×288 as the model input dimension.

Following the processing step of Droste et al. (2020b), MNetV2 is pre-trained with a large number of the processed US frames under the 14 SonoNet standard plane classifier (Baumgartner et al., 2017) to facilitate image representation learning. The raw gaze point used in this study is scaled to (−0.5,0.5) with the image center kept invariant, and the predicted $\mu_{t}^{OG}$ and $\mu_{t}^{MG}$ are also normalized to the same range by *sigmoid* activation and a shift factor 0.5 before the minimization of multi-task objective. In the training stage, we randomly select 32 continuous frames in each sample. Before prediction, we also allow 1s for processing the observed video frames by setting *t_0_* to 6 (Droste et al., 2020b). The model is evaluated for three typical standard planes of trans-ventricular plane (TVP), abdominal circumference plane (ACP), and femur standard plane (FSP) (Salomon et al., 2011) that are requested for fetal biometric measurements. The AdamW optimizer is adopted with an initial learning rate of 0.001 decayed by 0.01 every 8 epochs. The whole network is first trained on all 14 classes of standard planes for 20 epochs and separately fine-tuned for the three examined planes for 16 epochs.

We evaluate two scanning stages based on the probe position: *Coarse Adjustment* where probe rotation angle to standard plane >10°, and *Fine Adjustment* ≤10°. The ratio of the two stages may vary from sample to sample and thus prediction performance is averaged among all frames in the same stage. For our method, we randomly sample 100 trajectories from the predicted distribution and average them as a final prediction $\hat{r}$ and $\hat{s}$ as the numerical solution. In our approach, we investigate the training process of three policy combinations, each of which includes at least one local step action as training guidance following imitation learning, i.e., OP+OG (Section 4.1.1 and 4.2.1), SP+OG (Section 4.1.2), and OP+MG (Section 4.2.2). The optimization of the global policy combination (i.e., SP+MG) is a trial-and-error approach without relying on local demonstrations, which targets distinct clinical applications and will be considered for future investigation.

## 4 Results

### 4.1 Probe motion guidance

We measure the probe guidance accuracy based on the direction of rotation. This is because an accurate guidance will eventually lead to the target plane if it reduces the distance to the target orientation. With this observation, we assume a probe movement is correctly predicted if it is rotating towards the target plane, i.e., $∠(q_{t-1}{\hat{r}}_{t},q_{target})\leq \;∠(q_{t-1},q_{target}),$ with *q_target_* = *q_t_* for OP policy and *q_target_* = *q_T_* for SP. For each policy, we compare our multi-task model with the probe rotation baseline, i.e., *Baseline (r)*, continuing the previous probe rotation at the current time step, and the single-task architecture: *PoseReg*, image-based probe pose estimation approach using ResNet-18 (He et al., 2016) as adopted in (Di Vece et al., 2022); *US-GuideNet*, video-based probe guidance approach based on US image and previous probe movements (Droste et al., 2020b). In particular, *Baseline (r)* is to use the actual probe motion from the previous time step *r_t-1_* as the motion for the current time step, and it is used to compare with the motion ${\hat{r}}_{t}$ produced by predictors (PoseReg, US-GuideNet, or Multimodal-GuideNet) at the current time step.

#### 4.1.1 One-step probe prediction (OP)

**Fig. 3:**
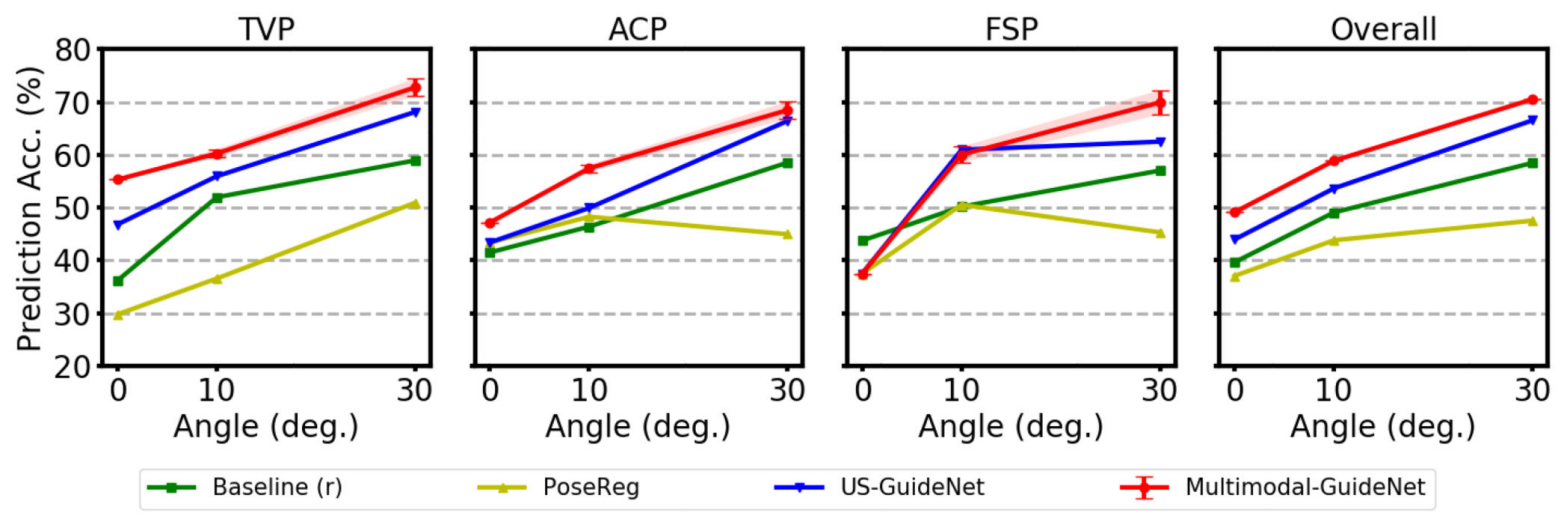
Performance of one-step probe guidance (OP) on the three evaluated standard planes and the overall prediction. The shaded area indicates the standard deviation of our model across all 100 samplings. The *x* axis is the rotation angle to the standard plane within the range of 30°.

The performance comparisons for the OP probe guidance policy are given in Fig. 3. Compared to the guidance method (PoseReg) that solely based on US image, both US-GuideNet and Multimodal-GuideNet leverage the temporal information from previous probe movements, leading to a more precise guidance of the next motion. Furthermore, Multimodal- GuideNet achieves an overall consistent improvement over the single task-based US-GuideNet (Droste et al., 2020b) for the two adjustment stages, which indicates that simultaneously learning the gaze patterns benefits the probe motion planning. In terms of biometric planes, the probe rotation for the femur (FSP) is difficult to predict for both single-task and multi-task models when it gets close to the standard plane (at 0°). Different from a steady probe movement to achieve TVP and ACP, the probe manipulation close to FSP sometimes requires complicated twisting actions (Salomon et al., 2011). This also explains why incorporating gaze contributes more in the coarse adjustment (as at 30°) to locate the femur but not in the fine stage (as at 10°). Moreover, the flexible movements of fetal limbs increase the diversity in FSP detection, which explains why there is a slightly higher standard deviation observed for this plane.

#### 4.1.2 Standard plane orientation prediction (SP)

**Fig. 4:**
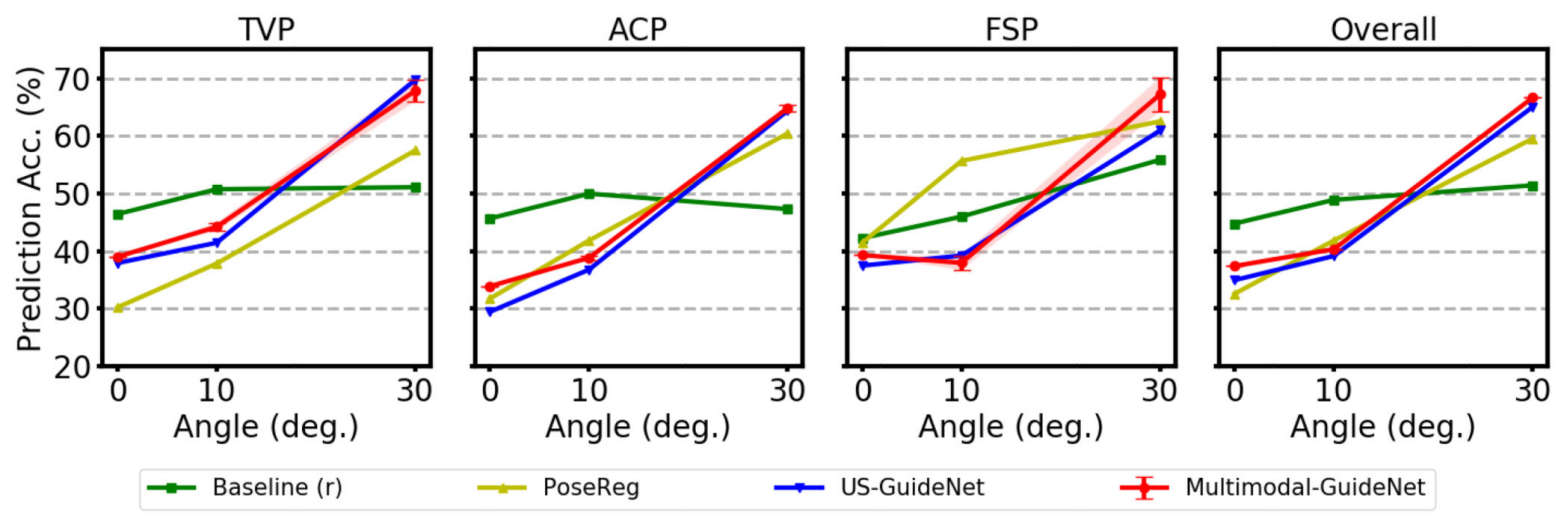
Performance of standard plane probe guidance (SP) on the three evaluated standard planes and the overall prediction.

The prediction accuracies under the SP guidance policy are compared in Fig. 4. The baseline, which follows the actual scanning path, outperforms other methods during the fine adjustment stage (as ≤10°) under the SP policy. This is because when under *coarse* adjustment, the actual rotation is more likely towards the standard plane which is consistent with the predicted rotation. While under the *fine* stage, the actual probe movement approaching the standard plane may contain frequent forward and backward rotations to finalize the best position, which is not always rotating toward the standard plane as indicated by US-GuideNet or Multimodal-GuideNet. Different from OP where the probe movements can be estimated from the observed frames, the standard plane position is anonymous to the model which also increases the uncertainty of the prediction.

We then observe that under the new policy, Multimodal- GuideNet outperforms US-GuideNet (Droste et al., 2020b) especially when close to standard plane (as at 0°). This may due to the fact that gaze is a short-term signal that is more informative for the probe refinement to lead to the SP. When increasing the angular distance, the two models perform comparably especially for TVP and ACP.

For femur, motion-informed methods perform worse than purely image-based PoseReg in finalizing the FSP, because the probe undergoes frequent adjustments in various directions that could introduce more degrees of freedom compared to the two femur ends defined on the image plane. However, with similar observations as in OP policy, Multimodal-GuideNet shows substantial improvement during the coarse stage when incorporating gaze for probe guidance. This may be due to only one certain structure in FSP compared to the other two planes, where the gaze pattern is more stable in searching the femur.

### 4.2 Gaze trajectory prediction

For the OG policy, the predicted gaze point ${\hat{g}}_{t}=g_{t-1}+{\hat{s}}_{t}$ is evaluated by pixel *ℓ_2_* norm error. Similar to the probe guidance, we compare with the following two architectures: *Base-line (g)*, using the actual gaze movement from the previous time step *s_t-1_* as the gaze motion for the current time step; *Gaze-GuideNet*, single-task learning approach for gaze prediction, where only gaze information is modeled and predicted from US video by discarding the probe stream from Multimodal-GuideNet. As a common evaluation in sampling-based generative models (Gupta et al., 2018), the performance of the best gaze point prediction among all samplings is also reported in Gaze-GuideNet* and Multimodal-GuideNet*, respectively. To validate the generated eye-tracking heat map, we also consider the commonly evaluated saliency map metrics (Bylinskii et al., 2018): the distribution-based metrics Similarity (SIM), Linear Correlation Coefficient (CC), and Kullback-Leibler divergence (KLD), and the fixation point-based metrics Area Under the ROC Curve by Judd (AUC) and Normalized Scanpath Saliency (NSS).

In MG, we set the time window length *F* to 5 which consists of about 1s of gaze movement pattern for multi-step prediction. Defined by the number of components in GMM, the number of gaze clusters *L* within the time window is set to 3, as empirically 1s of gaze motion contains no more than 3 fixations for image reading (Bylinskii et al., 2018). The predicted gaze centers ${\hat{c}}_{t}^{l}=c_{t-f}^{l}+{\hat{s}}_{t}^{l},l\leq 3$ are evaluated with distribution-based metrics SIM and CC. SIM measures the pixel-wise minimum value between the saliency map constructed from the predicted gaze GMM and the empirical saliency map based on the real gaze points. As opposed to the *ℓ_1_*-based SIM, CC depends on *ℓ_2_* norm that quantifies the correlation between two normalized saliency maps (Kummerer et al., 2018). On each plane, the averaged scores for each sequence are returned by averaging across time steps in each evaluation stage.

### 4.2.1 One-step gaze shift prediction (OG)

**Fig. 5:**
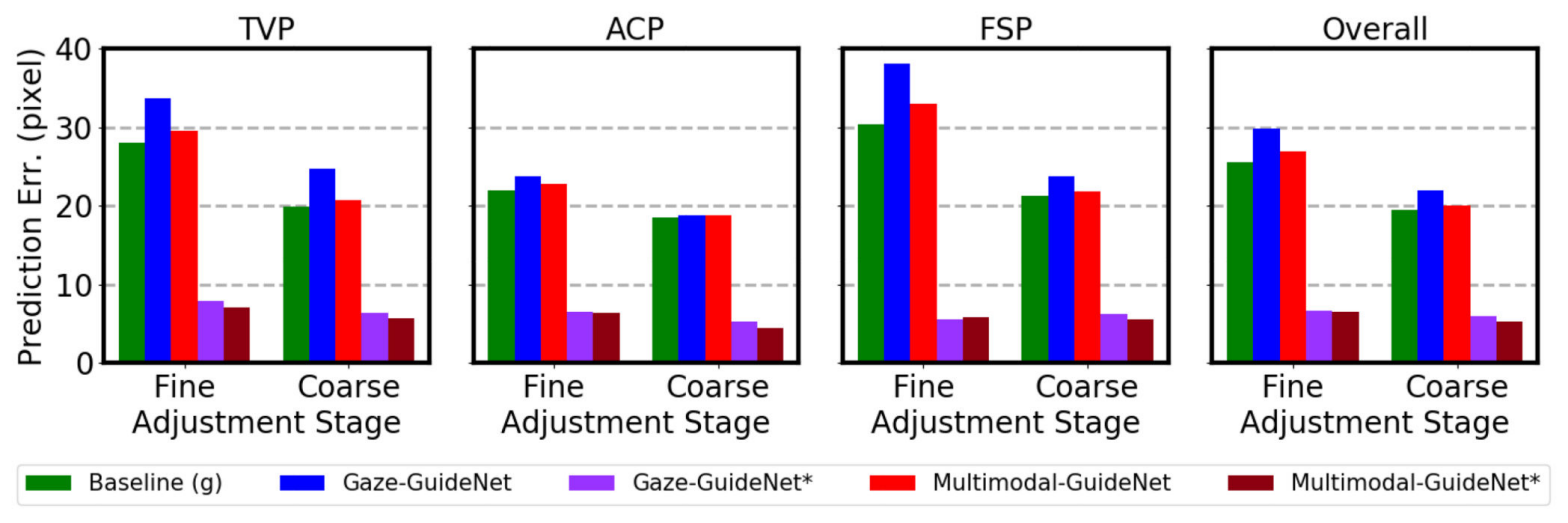
Gaze prediction error (the lower the better) on the three evaluated standard planes and the overall prediction. The error of the best-generated gaze point that is closest to ground truth is reported in Gaze-GuideNet* and Multimodal-GuideNet*, respectively.

Figure 5 shows the prediction results for the gaze task. A common observation among all three planes is that for gaze prediction, the error of fine adjustment is generally larger than coarse adjustment. This is because in contrast to the fine-grained probe motion, eye gaze movement during that time is quite rapid, flitting between the observed anatomical structures. When comparing the three planes, the error ranges are the lowest for ACP and the highest for FSP, especially for the fine adjustment. Since the key anatomical structures in the abdomen are relatively close to each other, the sonographer requires a smaller change in gaze. For FSP, sonographers switch focus between both femur ends that are relatively far from each other, which may increase the uncertainty of the gaze position in the next time step.

Comparing between methods, Multimodal-GuideNet reduces the error of Gaze-GuideNet for all cases, which demonstrates the effectiveness of multi-task learning over single-task learning in gaze prediction. The errors of Gaze-GuideNet* and Multimodal-GuideNet* are both within 10 pixels which shows the feasibility of the learned distribution in generating a plausible gaze trajectory. Practically, Multimodal-GuideNet* could be useful when a precise gaze is needed such as when the sonographer focuses on a small range of underlying anatomical structure, and its improvement over Gaze-GuideNet* indicates probe guidance could potentially help locate such a fixation point.

**Fig. 6:**
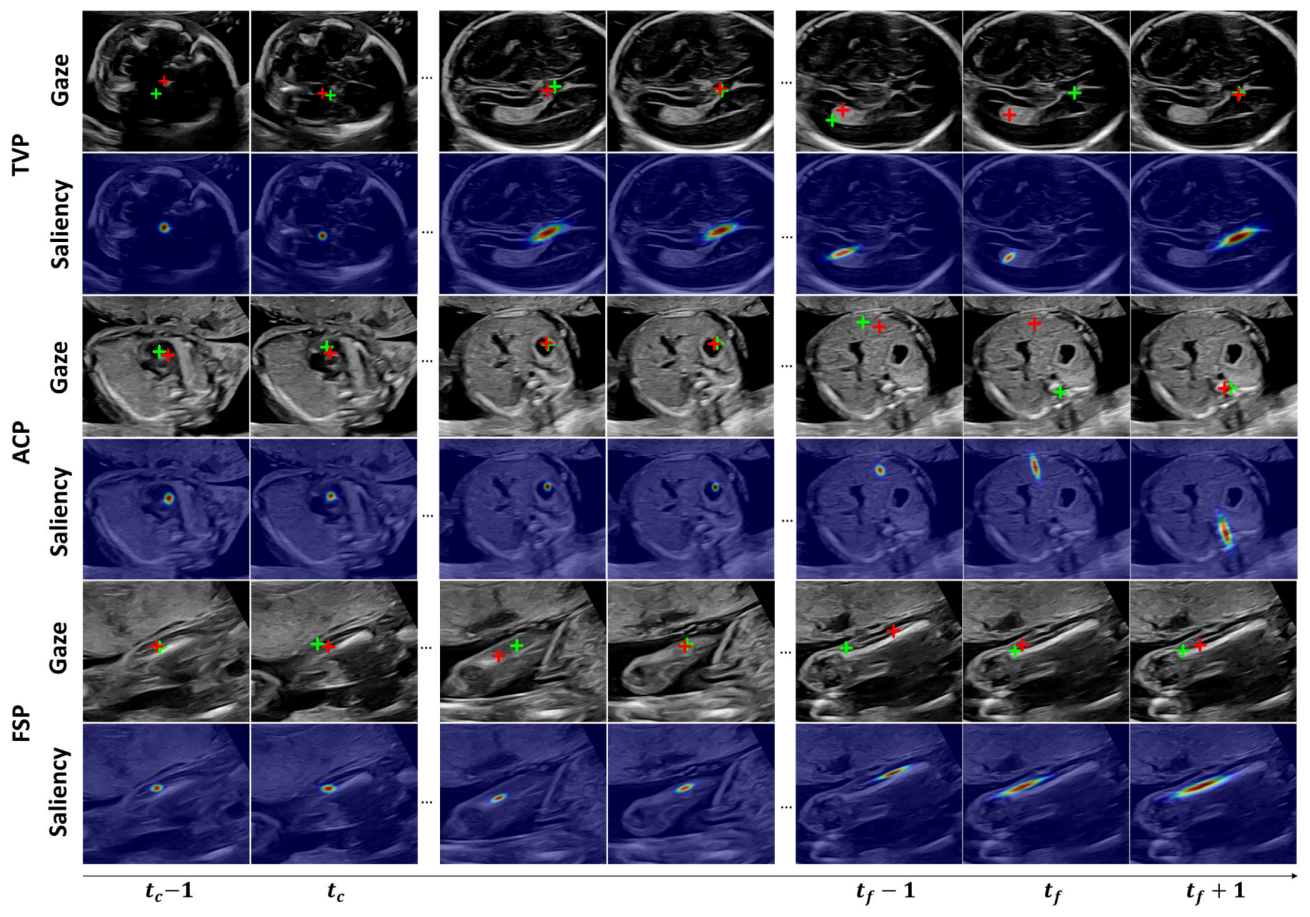
Visualization of predicted one-step gaze point (red cross), saliency map, and corresponding ground truth one-step gaze point (green cross) for TVP, ACP, and FSP searching sequences. *t_c_* and *t_f_* are timestamps for coarse and fine adjustment, respectively.

Figure 6 shows example sequences of the three planes with the predicted visual saliency and gaze point deduced from the generated gaze shift distribution. The predictions are highly accurate in all timestamps except for sometimes a significant gaze shift at fine adjustment steps (e.g. TVP and ACP at frame *t_f_*) where more transitionary gaze behaviour emerges to search between the anatomical structures. However, the predicted saliency map at those time steps correctly estimates the orientation of gaze shift. From *t_c_* to *t_f_*, the scale of the gaze saliency determined by $\sigma_{t}^{OG}$ generally increases, which also reflects the growing complexity of gaze movements when approaches the end of searching. For example, the saliency in FSP tends to cover more area in the femur where the gaze might occur.

The quantitative results with saliency map-based metrics are also compared in Table 1, where the multi-task model gener-ally outperforms the single-task one. For this one-step policy (OG), Multimodal-GuideNet also achieves an overall lower standard deviation across all saliency metrics, and the differences in mean scores hold statistical significance. It is also consistent with Fig. 5 that the average prediction in coarse stage provides more accurate distributions than the fine stage. In general, modeling the gaze information as a bi-variate distribution is technically advantageous over a saliency map-based predictor, as the problem complexity is reduced from optimizing a large feature map to only a few parameters for probability density estimation. The flexibility in gaze sampling also preserves the variety in gaze movements.

### 4.2.2 Multi-step gaze center prediction (MG)

For the MG policy, we evaluate how the predicted gaze centers are correlated with the real gaze distribution. Different from a single retrieved region, multiple areas are highlighted under MG policy by modeling the multi-step gaze distribution as a Gaussian mixture distribution. Table 2 shows the saliency scores of Multimodal-GuideNet with different numbers of predicted centers. The saliency prediction for coarse adjustment is overall better than that for the fine stage. Compared to OG, the values of saliency metrics under MG are lower since predicting multisteps ahead is more challenging with the upcoming US images unknown to the model.

The performance across the three planes also indicates some potential gaze moving patterns of expert sonographer. For TVP and ACP, the saliency prediction under two gaze centers (*L* = 2) usually performs the best, which indicates that two main locations are likely to be examined at the same time for a sonographer to adjust the probe to reach a good plane in a 1s time interval. This is similar to the observation in Teng et al. (2022) where the sonographer observes multiple structures for the abdominal and brain planes. The results of hypothesis testing also reveal that the two-gaze center (*L* = 2) exhibits a statistically significant differentiation from the other two clusters. The only ambiguity emerges in the fine phase of TVP, where distinguishing between *L* = 2 and 3 becomes demanding. This can be explained by the fact that the anatomies are visually close, such as mid-line echo and cavum septum pellucidum, and they will be naturally recognized as a single cluster when looking at these structures in the image plane. This also applies to ACP, particularly during the coarse stage, where the anatomical structures inside the abdomen are in close proximity presented on the image plane. While for FSP, the best prediction for the coarse stage is for one gaze center, which maybe due to that compared to the head and abdomen, the femur has relatively fewer structures to focus on during the beginning of the scan.

**Fig. 7:**
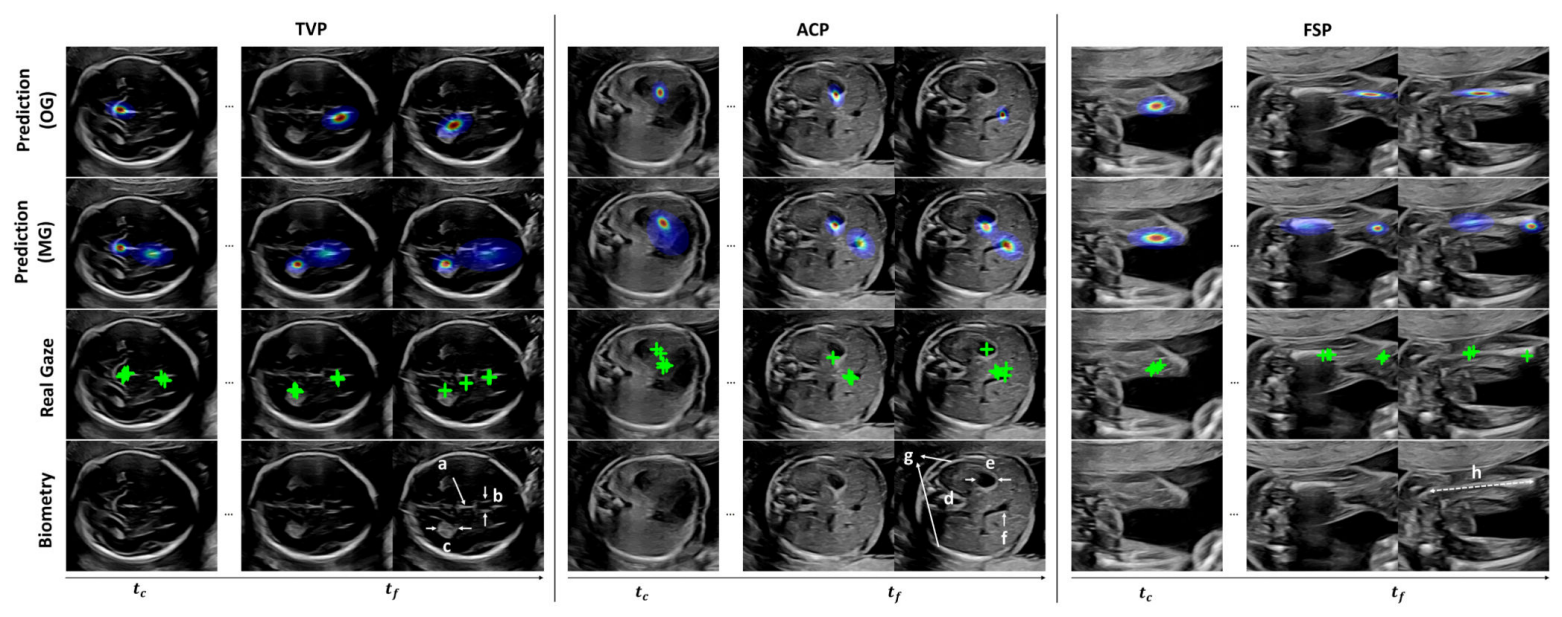
Qualitative comparison of predicted one-step (OG, 1^st^ row) and multi-step (MG, 2^nd^ row) saliency map for TVP, ACP, and FSP searching sequences. The corresponding ground truth multi-step gaze points (green cross, 3^rd^ row) and the video frames with key biometric landmarks (4^th^ row) are also given as reference. The key structures are (a) mid-line echo, (b) cavum septum pellucidum, (c) choroid plexus for TVP, (d) spine, (e) stomach bubble, (f) umbilical vein, (g) rib for ACP, and (h) femur for FSP.

Figure 7 visually compares the gaze saliency prediction under different policies. Unlike OG that only retrieves the most likely region, the predicted gaze centers under MG cover more anatomical structures that the sonographer may potentially focus on in the following scanning steps. Consistent with the cluster selection results in Table 2, for each biometry plane in the example sequence, two gaze centers are mainly retrieved within 1s of scan, which is consistent with the ground truth clusters. The spatial locations of gaze center predictions are also aligned with the corresponding anatomy. Specifically, the predicted gazes for TVP are initially located in the midline, and then move between the choroid plexus (CP) and cavum septum pellucidum (CSP) to allow the sonographer to accurately measure the fetal head. Compared to the head, the structures for the abdomen are closely distributed which explains why single fixation center (*L* = 1) of ACP also performs well in Table 2. For FSP searching, MG is capable to retrieve two sides of the femur where most fixations appear for measuring the femur length (Chudleigh et al., 2016).

### 4.3 Mechanism of modeling gaze and probe signals

**Fig. 8:**
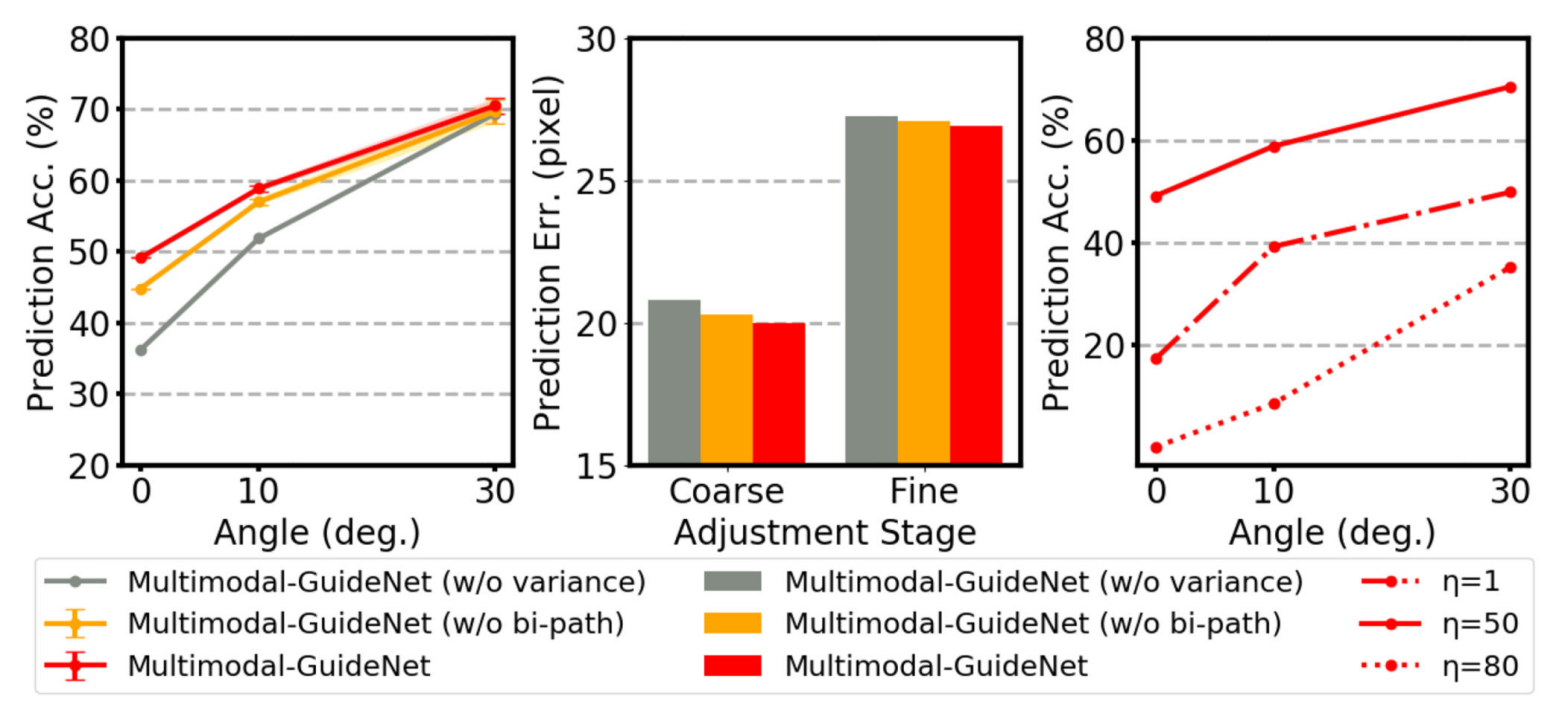
Evaluation for model design of bidirectional pathway between the gaze and probe movement (denoted as *bi-path*) and random sampling of the two signals (denoted as *variance)* under OP (left subplot) and OG (middle subplot), and the quaternion weight *η* under OP policy (right subplot).

Figure 8 showcases the quantitative results of the bidirectional pathway and the random variable modeling under the two tasks of one-step gaze prediction and probe guidance, and the hyper-parameter of the quaternion weight η under the probe guidance task. In the left subplot, the improvements (w/ vs. w/o bi-path) indicate that the pathway between gaze and probe stabilizes the probe movement with a more accurate prediction, especially in fine adjustment. The bidirectional pathway also slightly improves the gaze prediction as compared in the middle subplot.

When comparing Multimodal-GuideNet with and without variance, we find that modeling the gaze and probe signals as random variables substantially increases the performance of two-way guidance. It indicates that random sampling of the two signals not only allows scanning variations, but is also more accurate than predicting a fixed position in the obstetric scanning guidance to reach the desired plane.

We also evaluate different scales of the quaternion weight in the right subplot of Fig. 8. The value of η that yields the highest prediction accuracy is selected as 50. Given that the quaternion prior is a hard constraint, it is assigned a much higher weight (η = 50) than the two guidance losses (*λ_s_* = *λ_r_* = 1) to ensure that the probe prediction exhibits rotational properties, and an improper choice of η can lead to an incorrect estimation of the probe rotation.

### 4.4 Sensitivity analysis

To test the reliability of Multimodal-GuideNet, we run the model three times with different training seeds for random parameter initialization, and it obtains a standard deviation of 0.04°for probe rotation prediction and 0.27 pixel for gaze estimation. The minor biases show the robustness of the proposed Multimodal-GuideNet in producing consistent predictions during each execution, thus enhancing the confidence in its clinical applications.

## 5 Conclusion and discussion

This paper presents a novel multimodal framework for the joint guidance between probe motion and eye-tracking data in routine US scanning. We have explored multi-task learning by jointly predicting the probe rotation and gaze trajectory from US video via a shared modality-aware graph structure. During inference, we explore two policies for probe guidance: 1) OP policy, following the human sonographer performing the scan that provides stepwise guidance gradually approaching the standard plane, and 2) SP policy, simulating machine scan that directly moving towards the standard plane. Under both policies, we show the effectiveness of jointly learning multi-modality data over single-modality-based modeling. In terms of gaze, we also improve our previous work from predicting only single saliency region (OG policy) to multiple regions (MG policy) in the image plane that expert sonographers may be interested in, and the best-retrieved saliency areas are approximately matched with the positions of anatomical landmarks in obstetric scans. The performance gains over single-task predictions suggest that the two-modality signals complement each other to reach the scanning target, while ignoring either of them will lead to a biased guidance.

The results presented in Section 4.1 illustrate that the probe guidance of femur scanning generally achieves a higher error compared to the abdomen and head. Because of the unfixed position of fetal limbs relative to the body, it may cause more unpredictable probe motions compared to other measurements. The gaze guidance under MG policy is clinically more applicable than OG since usually more than one anatomical structure would be evaluated in a fetal biometric plane. According to the result of MG policy in Section 4.2.2, the optimal strategy is to regress two visual centers, which follows the clinical practice that the experienced sonographer will probably examine on average two anatomical structures at the same time when searching the biometric view.

The learned two-way guidance signals with random sampling also allow for diversity between individual scans in a practical environment. Furthermore, by modeling the Gaussian distribution and GMM for gaze prediction, the two policies OG and MG will each keep the minimum number of prediction parameters to avoid an exhausted generation of the whole image-sized saliency map. This is because the inference time is also a key factor for real-time scanning, and a lightweight guidance model is preferred with not only accurate prediction but also a prompt response.

Practically, understanding the causal relationship between ultrasound multi-modality signals can enhance training skills for medical professionals. Trainee sonographers can receive immediate feedback on their visual focus, their techniques for manipulating the probe, and the corresponding impact on ultrasound image quality. With proper probe and gaze guidance, high-quality image planes can be obtained for precise measurements of fetal conditions. Additionally, fewer unnecessary movements could also lead to more efficient ultrasound procedures and a better experience for pregnant women. In this paper, we provide multimodal ultrasound scanning guidance towards fetal biometric measurements, and we expect the experimental observations could shed light on the research of understanding multi-modality dependencies in medical screening.

## Figures and Tables

**Table 1:** Results of visual saliency comparison between single modality Gaze-GuideNet (gaze only) and Multimodal-GuideNet (gaze+probe). Best values are marked bold. ↑ the higher the better, and ↓ the lower the better. † denotes an improvement of Multimodal-GuideNet with statistical significance (p < 0.05) over Gaze-GuideNet.

Stage	Architecture	SIM ↑	CC ↑	KLD ↓	AUC ↑	NSS ↑
Coarse	Gaze-GuideNet	0.447 ± 0.01	0.626 ± 0.04	1.711 ± 0.04	**0.774** ± 0.05	1.542 ± 0.53
	Multimodal-GuideNet	**0.462** ± 0.01†	**0.634** ± 0.03†	**1.665** ± 0.02†	0.766 ± 0.06	**1.866** ± 0.37†
Fine	Gaze-GuideNet	0.341 *±* 0.01	0.539 ± 0.02	2.413 ± 0.04	0.780 ± 0.03	1.544 ± 0.26
	Multimodal-GuideNet	**0.350** ± 0.01†	**0.543** ± 0.02†	**2.093** ± 0.02†	**0.785** ± 0.03†	**1.871** ± 0.29†

**Table 2:** Performance of the number of predicted gaze centers (*L*) against the three examined planes under different probe adjustment stages. † and ‡ denote an improvement with *p <* 0.05 of the two gaze centers (*L* = 2) over one (*L* = 1) and three centers (*L* = 3), respectively.

Stage	*L*	TVP		ACP		FSP	
		SIM	CC	SIM	CC	SIM	CC
Coarse	1	0.323 ± 0.01	0.435 ± 0.03	0.378 ± 0.01	**0.494** ± 0.03	**0.313** ± 0.01	**0.411** ± 0.02
	2	**0.347** ± 0.01†‡	**0.441** ± 0.03†‡	**0.383** ± 0.01†‡	0.484 ± 0.04†‡	0.299 ± 0.01†‡	0.356 ± 0.04†‡
	3	0.331 ± 0.01	0.407 ± 0.03	0.363 ± 0.01	0.449 ± 0.04	0.285 ± 0.01	0.324 ± 0.03
Fine	1	0.270 ± 0.01	0.365 ± 0.02	0.327 ± 0.01	0.443 ± 0.02	0.274 ± 0.01	0.375 ± 0.02
	2	**0.304** ± 0.01†	**0.383** ± 0.01†‡	**0.346** ± 0.01†‡	**0.451** ± 0.02†‡	**0.332** ± 0.01†‡	**0.425** ± 0.02†‡
	3	0.303 ± 0.01	0.374 ± 0.03	0.336 ± 0.01	0.434 ± 0.03	0.329 ± 0.01	0.407 ± 0.02

## References

[R1] Baumgartner CF, Kamnitsas K, Matthew J, Fletcher TP, Smith S, Koch LM, Kainz B, Rueckert D (2017). Sononet: real-time detection and localisation of fetal standard scan planes in freehand ultrasound. IEEE Trans Med Imaging.

[R2] Bylinskii Z, Judd T, Oliva A, Torralba A, Durand F (2018). What do different evaluation metrics tell us about saliency models?. IEEE transactions on pattern analysis and machine intelligence.

[R3] Cai Y, Sharma H, Chatelain P, Noble JA (2018a). Multi-task sonoeyenet: detection of fetal standardized planes assisted by generated sonographer attention maps.

[R4] Cai Y, Sharma H, Chatelain P, Noble JA (2018b). Sonoeyenet: Standardized fetal ultrasound plane detection informed by eye tracking.

[R5] Cho K, van Merrienboer B, Gulcehre C, Bougares F, Schwenk H, Ben-gio Y (2014). Learning phrase representations using rnn encoder-decoder for statistical machine translation.

[R6] Chudleigh T, Smith A, Cumming S (2016). Obstetric & Gynaecological Ultrasound.

[R7] Di Vece C, Dromey B, Vasconcelos F, David AL, Peebles D, Stoyanov D (2022). Deep learning-based plane pose regression in obstetric ultrasound. International Journal of Computer Assisted Radiology and Surgery.

[R8] Droste R, Cai Y, Sharma H, Chatelain P, Drukker L, Papageorghiou AT, Noble JA (2019). Ultrasound image representation learning by modeling sonographer visual attention.

[R9] Droste R, Chatelain P, Drukker L, Sharma H, Papageorghiou AT, Noble JA (2020a). Discovering salient anatomical landmarks by predicting human gaze.

[R10] Droste R, Drukker L, Papageorghiou AT, Noble JA (2020b). Automatic probe movement guidance for freehand obstetric ultrasound.

[R11] Drukker L, Sharma H, Droste R, Alsharid M, Chatelain P, Noble JA, Papageorghiou AT (2021). Transforming obstetric ultrasound into data science using eye tracking, voice recording, transducer motion and ultrasound video. Sci Rep.

[R12] Graves A (2013). Generating sequences with recurrent neural networks.

[R13] Grimwood A, McNair H, Hu Y, Bonmati E, Barratt D, Harris EJ (2020). Assisted probe positioning for ultrasound guided radiotherapy using image sequence classification.

[R14] Guo H, Xu S, Wood B, Yan P (2020). Sensorless freehand 3d ultrasound reconstruction via deep contextual learning.

[R15] Gupta A, Johnson J, Fei-Fei L, Savarese S, Alahi A (2018). Social gan: Socially acceptable trajectories with generative adversarial networks.

[R16] He K, Zhang X, Ren S, Sun J (2016). Deep residual learning for image recognition.

[R17] Housden RJ, Treece GM, Gee AH, Prager RW (2008). Calibration of an orientation sensor for freehand 3d ultrasound and its use in a hybrid acquisition system. Biomed Eng.

[R18] Kay A (2007). Tesseract: an open-source optical character recognition engine. Linux Journal.

[R19] Kummerer M, Wallis TS, Bethge M (2018). Saliency benchmarking made easy: Separating models, maps and metrics.

[R20] Li K, Wang J, Xu Y, Qin H, Liu D, Liu L, Meng MQH (2021). Autonomous navigation of an ultrasound probe towards standard scan planes with deep reinforcement learning.

[R21] Li Y, Zemel R, Brockschmidt M, Tarlow D (2016). Gated graph sequence neural networks.

[R22] Men Q, Teng C, Drukker L, Papageorghiou AT, Noble JA (2022). Multimodal-guidenet: Gaze-probe bidirectional guidance in obstetric ultrasound scanning.

[R23] Mustafa ASB, Ishii T, Matsunaga Y, Nakadate R, Ishii H, Ogawa K, Saito A, Sugawara M, Niki K, Takanishi A (2013). Development of robotic system for autonomous liver screening using ultrasound scanning device.

[R24] Prevost R, Salehi M, Jagoda S, Kumar N, Sprung J, Ladikos A, Bauer R, Zettinig O, Wein W (2018). 3d freehand ultrasound without external tracking using deep learning. Med Image Anal.

[R25] Salomon LJ, Alfirevic Z, Berghella V, Bilardo C, Hernandez-Andrade E, Johnsen S, Kalache K, Leung KY, Malinger G, Munoz H (2011). Practice guidelines for performance of the routine mid-trimester fetal ultrasound scan. Ultrasound Obstet Gynecol.

[R26] Sandler M, Howard A, Zhu M, Zhmoginov A, Chen LC (2018). Mo-bilenetv2: Inverted residuals and linear bottlenecks.

[R27] Sharma H, Drukker L, Chatelain P, Droste R, Papageorghiou AT, Noble JA (2021). Knowledge representation and learning of operator clinical workflow from full-length routine fetal ultrasound scan videos. Medical Image Analysis.

[R28] Teng C, Sharma H, Drukker L, Papageorghiou AT, Noble JA (2021). Towards scale and position invariant task classification using normalised visual scanpaths in clinical fetal ultrasound.

[R29] Teng C, Sharma H, Drukker L, Papageorghiou AT, Noble JA (2022). Visualising spatio-temporal gaze characteristics for exploratory data analysis in clinical fetal ultrasound scans.

[R30] Toporek G, Wang H, Balicki M, Xie H (2018). Autonomous image-based ultrasound probe positioning via deep learning.

[R31] Wang S, Housden J, Noh Y, Singh D, Singh A, Skelton E, Matthew J, Tan C, Back J, Lindenroth L (2019). Robotic-assisted ultrasound for fetal imaging: evolution from single-arm to dual-arm system.

[R32] Yan S, Xiong Y, Lin D (2018). Spatial temporal graph convolutional networks for skeleton-based action recognition.

[R33] Zhang P, Lan C, Zeng W, Xing J, Xue J, Zheng N (2020). Semantics-guided neural networks for efficient skeleton-based human action recognition.

[R34] Zhao C, Droste R, Drukker L, Papageorghiou AT, Noble JA (2021). Visual-assisted probe movement guidance for obstetric ultrasound scanning using landmark retrieval.

[R35] Zhao C, Droste R, Drukker L, Papageorghiou AT, Noble JA (2022). Uspoint: Self-supervised interest point detection and description for ultrasound-probe motion estimation during fine-adjustment standard fetal plane finding.

[R36] Zhao H, Wildes RP (2021). Where are you heading? dynamic trajectory prediction with expert goal examples.

